# Round the Hospitals

**Published:** 1920-06-19

**Authors:** 


### ROUND THE HOSPITALS.

The King held Investitures at Buckingham Palace on June 8 and 11, when His Majesty conferred decorations on members of the nursing services as follows:—Royal Red Cross, First Class: Miss Emma Dodd, T.F.N.S., and Mrs. Sarah O'Keefe, B.R.C.S.; Misses Elizabeth Baillie, Marion Barwell, Ida Brooke and Maud Plaskitt, Q.A.I.M.N.S.R.; Misses Mary Bate, Gertrude Bulman, Ada Peppier, and Annie Leech, T.F.N.S.; Miss N. MacMillan, C.N.S.; Mrs. May McWalters, V.A.D. Military Medal: Miss Beatrice Dascombe, Miss Maude de Guerin, and Miss Marie McGrath,

**ROUND THE HOSPITALS**—*(continued).*

Q.A.I.M.N.S.R.; Miss Katherine Lowe, T.F.N.S. Royal Red Cross, Second Class: Miss Nellie Thompson, Civil Nursing Service; Miss Helen Montfort, B.R.C.S.; Misses E. Bayley, E. de Trafford, and J. Gaydon, V.A.D.; Miss Eliza Covey, S.A.A.M.C.; Misses Lilian Newland and Jane Young, Q.A.I.M.N.S.R.; Misses Mary Brown, Jessie Morty, Ada Murray, Ann O'Donnell, Daisy Perry, and Evelyn Pike, Q.A.I.M.N.S.R.; Misses Violet Beamish, Lucy Bowman, Beatrice Brayshaw, Margaret Briggs, and Annie Fishwick, T.F.N.S.; Miss Julia Armstrong, C.N.S.; Mrs. Maud Burridge, Misses Amelia Cargill, Barbara Jeffreys, Sarah Norfield, Alice Phillips, and May Purdle, B.R.C.S.; Mrs. Edith Faunce, Misses Rosamund le Cocq, and Daisy Russell, V.A.D. On the occasion of both Investitures Queen Alexandra graciously received the members of the nursing services who had received decorations, together with Miss A. B. Smith, at Marlborough House after the ceremony. We rejoice to hear that Her Majesty is now making rapid progress towards recovery.

---

A simple but touching ceremony marked the unveiling by the Duchess of Albany on June 11 of a tablet over a cot in her own ward, at the Royal Hospital for Women and Children, Waterloo Bridge, in memory of the work of Voluntary Aid Detachment nurses in the war. The Archbishop of Canterbury officiated at a short dedication, and spoke of the strength and energy, the love and devotion to the service of others commemorated in this offering of a memorial cot—the right kind of thing to keep in mind the stress and strain, the high hopes and achievements of those devoted women. Then the Duchess came forward and drew aside the veil. The tablet was inscribed "V.A.D. Cot, in memory of the work done by the V.A.D.s who served in this hospital during the great war, 1914-1919." The first patient, a bright little girl called Ellen French, was already in occupation and well enough to enjoy the focus of attention on her little bed and a chat with the Duchess and Archbishop. A bouquet of pink and white carnations was presented to the Duchess before she left.

---

The thirty-third annual general meeting of the members of the Royal National Pension Fund for Nurses will be held at the Royal Society of Arts, John Street, Adelphi, W.C., on Wednesday, June 23, at 4 P.M. The report to be presented shows an increase in the number of policies issued during 1919 as compared with 1918, the figures being respectively 754 policies, assuring immediate annuities of £860, and deferred annuities of £10,688, as against 603 policies assuring immediate annuities of £345, and deferred annuities of £8,581. The total premiums received in 1919 amounted to £97,563, an increase of over £3,500, compared with 1918. The total number of nurses drawing annuities at the end of 1919 was 2,723, the average annuity being slightly over £26 8s. The invested funds were increased during the year by £25,161, the total amount invested being £2,161,821. All interest earned above 3¾ per cent. was placed to the Investments Reserve Fund, which at the end of 1919 stood at £196,132.

---

During 1919 the Fund's Benevolent Branch, the Junius S. Morgan Benevolent Fund, received £583 in donations and subscriptions, in addition to £1,000 to be devoted to helping nurses to pay the premiums on their insurance when this was impossible owing to a breakdown in health. The grants made during the year amounted to £2,064, and to enable these grants to be made a sum of £525 was transferred from reserve fund. The committee feels there can be no reduction in their expenses in the near future, and begs for increased subscriptions, which are indeed necessary, seeing that the amount received in 1919 was insufficient by nearly £1,500 to meet the grants which it made. We are glad to note in the subscription list a sum of £60 from the sisters of the 2nd Eastern General Hospital, Dis. II., a generous gift which, we hope, may stimulate other hospital staffs in a like direction. If each Pension Fund nurse would give only 1s. a year to the Junius S. Morgan Benevolent Fund its difficulties would be considerably lightened, and its power for usefulness increased. This was a fact that the late Sir Henry Burdett never lost an opportunity of driving home to the members at the Pension Fund's annual meetings, and we venture to hope that they will this year resolve never in the future to miss adding this shilling to each yearly premium payment, at any rate, which they make to the Fund. The Council of the Fund, in reporting the death of Sir Henry Burdett, expresses its sense of very great loss owing to his unique knowledge of all matters relating to the nursing profession, which was of the utmost value to his colleagues.

---

We are requested to remind candidates for the College of Nursing and Cowdray Studentships that the entrance examination will be held on Saturday, June 26, at 10 A.M. to 1 P.M. and 2 P.M. to 5 P.M., at 7 Henrietta Street, Cavendish Square W. 1, and at 8 Drumsheugh Gardens, Edinburgh.

---

This is a month of conferences. The College of Nursing Conferences this week will be succeeded by the Nursing and Midwifery Exhibition to be held at the Royal Horticultural Hall from June 22 to 25, which will be marked by a very interesting series of papers and discussions at three separate sessions in the afternoon and evening on June 23 and 24. On the Wednesday, Mr. Briant will discuss "Living Out"; Miss Alderman is to speak on Public Health work, and other topics are mental and fever work and the difficulties of small hospitals. The Thursday is devoted to midwifery, infant welfare centres, and infant feeding, and the speakers include Dr. Gordon Lay, Miss Olive Haydon, Miss Neville (Middlesex Hospital), and Miss Liddiard. The exhibition promises unusual interest this year, for the

**ROUND THE HOSPITALS—(continued).**

Royal Free Hospital is displaying a model maternity and gynæcological ward, the Royal Ophthalmic will exhibit eye appliances, the Edmonton Military Hospital will show a fracture bed, the South Kensington Nurses' Co-operation a table drum with mask and lamp, and the Ministry of Health some specimen charts. Tickets will be sent free to nurses and midwives applying with stamped envelope to the Secretary, 22-24 Great Portland Street, W. A further interesting conference is that of the Incorporated Society of Trained Masseuses to be held on June 24, 25 and 26. The conference will include demonstrations. The complete programme is given on p. 312 of the present issue.

The building of a new and enlarged nurses' home for University College Hospital, now rendered possible by the munificent Rockefeller gift referred to elsewhere in this issue, is estimated to cost over £100,000. By a kind of prophetic instinct the site was acquired last autumn, and it is hoped to get the new building erected in three years' time. The transformation of the hospital, with the formation of the obstetric unit and the inauguration of numerous special departments, will profoundly affect the training-school for nurses quite independently of the change of quarters. University College Hospital has long been in the van of progress, and it may be anticipated that when all the new developments are in working order it will rank among the finest training-schools for nurses in the world.

Although the higher training of nurses is not specifically included in the objects of the Rockefeller donation, it is well known that this is a subject of great interest to the far-seeing philanthropists who control the Rockefeller Foundation. For some time past they have been inquiring into the facilities offered by the nurse-training schools for preparing women to undertake public health nursing. They have appointed a Standing Committee to deal with public health nursing education, which, in America, as in this country, is far from perfect. Last February the Rockefeller Foundation called a conference in New York to consider the best means of educating women for this important career. It was attended by a representative group of doctors, superintendents of nurses, and others; but, alas! the main point elicited was the shortage of candidates for the training-schools. Irrespective of the unattractive conditions attendant upon nurse-training, evidence was given to show that this shortage of students is now being felt in every profession and in every branch of work. The Rockefeller Committee is now investigating the entire system of nurse education in the States, and proposes to make a complete survey of the schools. The effect of this big undertaking is likely to be international, and it encourages us to hope that among the aims of the munificent donors to University College Hospital that of enlarging the scope of nursing education finds an appropriate place.

A very impressive scene took place in Paris on June 12 when the monument erected to the memory of Edith Cavell by the generosity of the French journal *Le Matin* was unveiled in the Tuileries Gardens. There was a large assembly of representatives of the French nation and of the Allies. Mr. Wainwright, nephew of Miss Edith Cavell, represented her family. The ceremony opened with the playing of the Marseillaise, followed by the British, Belgian, and American national anthems. The curtain was then drawn aside and the work of M. Gabriel Pech displayed. The monument consists of a white marble bas relief, twenty feet high, on which the figure of Miss Cavell is portrayed lying dead on the ground amidst the smoking ruins of a village. A German helmet rests on her lap and an angel hovers above scattering flowers. The design is perhaps hardly in line with the simple sincerity of the nurse, but it has been beautifully executed and adorns the fine site where it rests. Some eloquent speeches were made on the occasion, that of Lord Burnham, who spoke in French, being particularly happy. He spoke as one who for twenty years had been a member of the London Hospital Committee, and he testified to the fact that Edith Cavell was "just the true type of the London Hospital nurse." The text of his speech will be found on p. 311.

On the same day when Edith Cavell's memorial was being unveiled in Paris, Captain Canzio Garibaldi and Captain Domenico Palazzoli, who came for the purpose as representatives of the Italian Army, laid a wreath of flowers at the foot of the Edith Cavell Memorial facing Trafalgar Square. A very large crowd assembled to witness this graceful act of recognition and reverence. Members of all the Italian societies in London marched with their banners in procession to the statue, the two officers, each in his old uniform and carrying a flag, at the head. The wreath consisted of laurels with pink and white carnations on a background of white pinks. The inscription, in Italian and English, is as follows: "To the gentle English heroine whose sublime martyrdom has rendered the name of her fatherland sacred throughout the world. From Italian soldiers." Captain Palazzoli made a fine speech in which he declared that the manner in which Edith Cavell defied death places her side by side with the purest saints who died for the triumph of Christianity. Captain Garibaldi, speaking in Italian, made an earnest appeal for goodwill between the peoples of England and Italy.

The annual meeting of the Association of Hospital Matrons will be held on Saturday, June 19, in the Governors' Hall of St. Thomas' Hospital at 3.30 p.m. After the meeting Miss Lloyd Still will entertain the members to tea.

It is a happy sign of the close ties in aspiration and ideals which bind together the Poor-Law Nursing Services and the voluntary training-schools that the movement for introducing sister-tutors is

**ROUND THE HOSPITALS**—(*continued*).

steadily spreading to the municipal hospitals. The Paddington Guardians have recently appointed Miss M. Windley to be sister-tutor at the Paddington Poor-Law Infirmary at a salary of £100 a year, rising by yearly increments of £5 to £120, war bonus being merged. At the moment Miss Windley is in the midst of her course of training in the Household and Social Science Department of King's College for Women, and she will not take up her duties at the infirmary till next January. How many Poor-Law matrons who have broken down prematurely under the strain of controlling the nurses' education in addition to their overwhelming administrative responsibilities might now be in the exercise of their full powers had this relief been earlier available!

---

One of those perennial outbreaks of discontent which are liable to afflict institutions has occurred at the Bermondsey Infirmary and, as often happens when fairly investigated, proves difficult to account for. The Guardians spent eight hours recently trying to get to the bottom of various allegations and rumours. One by one the causes of resignations were patiently investigated. Everything seemed quite above board. The assistant medical officer had secured a better appointment. Three probationers had completed their course; one left from ill-health, and so on. A staff nurse left because the shortage of nurses threw too much work on her. Here was, at any rate, one loop-hole which let in light. But when it came to complaints about food and bed-clothes this very nurse declared the institution was the finest she had ever been in as regards the abundance and good quality of the food. The matron gave evidence to the same effect and decisively vindicated the bed-coverings. And the committee is left still investigating and decidedly puzzled.

We learn with great satisfaction that it is in contemplation to make some much needed concessions as regards workers' hours to the nursing staff at the Hospital for Sick Children, Great Ormond Street. The new matron, Miss Tisdale, has just come into residence and has the matter much at heart. There is no doubt that the ordinary demands on the nurse's time and attention are greatly augmented in the case of sick children and infants. The hours on duty at Great Ormond Street have long been recognised to be too long and the whole institution would greatly benefit by their reduction. The scheme for establishing an auxiliary hospital in the country is highly desirable, and the more widely the urgent need for this auxiliary is made known the quicker it should materialise. The mere fact that chronic cases often have to wait for a bed in a suitable home four or six months should open the hearts and purses of the rich mothers in the land.

---

The Council of the North Ormesby Hospital, Middlesborough, has decided to introduce a fifty-six-hour working week for the nurses, and has already made arrangements for the occupation by the extra probationers of a house adjacent to the hospital until a suitable nurses' home is provided. The hospital now contains 102 beds, having undergone nine extensions since its foundation in Dundas Mews, in 1858, as the first cottage hospital in England. The cost per patient rose from 7s. 2d. per day in 1918 to 8s. 6¾d. in 1919. In 1916 it was 5s. 4½d. Happily the receipts exceed those of any previous year owing to the splendid response made by the various employees to the House Committee's appeal.

---

The Committee of the Beckett Hospital, Barnsley, have made further increases in the salaries of the nursing staff, which now stand as follows: Matron "a substantial increase," assistant matron £100, massage sisters £90 to £100, night sisters £70 to £85, theatre sisters £65 to £80, staff nurses £55 to £60, probationers £20, £25, £30. The ward maids and other employees have all received an increase of pay. It is a highly satisfactory arrangement which leaves no one outside its scope.

---

The award of the Conciliation Board at the Cardiff Mental Hospital has resulted in a higher salary being allotted to some of the sisters than to the deputy-matron, whose case did not come under consideration by the Board. This curious anomaly was pointed out by the medical superintendent, Dr. Goodall, to the Hospital Committee, and has now been satisfactorily rectified. The Conciliation Board awarded an all-round increase of 8s. a week to each member of the nursing staff and 6s. a week to each domestic. No one can deny that the staff are well earning their extra £20 a year.

---

Another institution has been added to those of the Edith Cavell Homes of Rest for Nurses, by the opening of the freehold house at The Hollies, Gipsy Road, West Norwood. The Home, which is under the superintendence of Miss Mabel Foreman, R.R.C., is capable of receiving ten nurses at a time. A reception at The Hollies is being given by Miss Foreman on the 24th inst., from 3 to 6 p.m., when friends and members of the nursing profession will be welcomed and shown over the establishment.

---

Nurse Hillier, who sued the Tottenham District Council for damages for wrongful dismissal six months ago, has gained her case. It will be remembered that Nurse Hillier was engaged by the Tottenham District Council as nurse in their Maternity and Child Welfare Centre. She was dismissed at a month's notice by the committee of this Centre, but without confirmation from the District Council. Judge Crawford ruled, when the case came before him on June 10 at the Edmonton County Court, that there had been no effective ratification by the Council of the action of the committee, and he gave judgment for the plaintiff for £82 15s., the amount of six months' salary and bonus. Notice of appeal was given.

